# Discouraging the Use of Tobacco

**Published:** 1914-06

**Authors:** 


DISCOURAGING THE USE OF TOBACCO.—A great deal is being said in the medical papers, as well as in the newspapers, about the evil effects of the use of tobacco upon boys and young men. So far as opinions have been published, there appears to be but one side to the question; tobacco, and especially cigarettes, dull the brain and retard the development of the mental faculties of the boys, and its use is therefore discouraged by all medical experts, and by all employers of young men. Teachers in the public schools and colleges, without exception, testify that smoking incapacitates the student for best work.

The surprising thing is that but little has been said about the evil effects of tobacco upon the mature man. It stands to reason that the use of anything that will injuriously affect the nerve system and consequently the brain of a young person will in some measure at least have the same effect upon older persons. It may be because so many members of the medical profession, so many preachers, so many teachers, and so many others are addicted to the use of tobacco and dislike to give it up themselves, that they have failed to see or to study its bad effects upon any others than the young. We should be honest with ourselves and, if tobacco is harmful, should stop its use.

At a recent meeting of the general conference of the Methodist Church a decision was reached not to receive into the ministry any one addicted to the use of tobacco in any form, but, strange to say, not one word was said by the conference against the use of tobacco on the part of those already in the ministry. This looks like an inconsistent attitude, but it is no more inconsistent than advice given by users of tobacco to young men to abstain from its use. The time is coming, and is coming soon, when the use of tobacco in any form will be regarded, not only as a useless and nasty habit, but as one injurious especially to those who depend largely upon mental work. The fact that a great many of our large business concerns do not now employ men who smoke will cause many others to see the advantages to be derived from abstaining from tobacco altogether. Smoking has for a long while been somewhat of a fad among students in our higher institutions of learning, but, if they come to know that the habit retards them in their work, it will doubtless become unpopular, and the students who will then use tobacco will be few indeed.

Physicians can do much to stop tobacco using, but they can accomplish little in this direction so long as they attempt to show the evil effects of tobacco upon the brain while they themselves are constant smokers. Physicians are expected, not only to teach the things that are best for public health, but also to set examples by the practice of their own teaching, and gradually more and more of the best physicians will come to abandon the use of tobacco, just as practically all of them have already quit the use of intoxicating liquors.

